# NOVEL GAMMA-SECRETASE MODULATOR REGULATES APP PROCESSING AND INFLAMMATORY RESPONSES IN NPM-EXPOSED MICE

**DOI:** 10.1093/geroni/igz038.353

**Published:** 2019-11-08

**Authors:** Carla D'Agostino, Mafalda Cacciottolo, Constantinos Sioutas, Steven L Wagner, Rudolph E Tanzi, Todd E Morgan, Caleb E Finch

**Affiliations:** 1 University of Southern California, Los Angeles, California, United States; 2 Viterbi School of Engineering, University of Southern California, Los Angeles, California, United States; 3 Department of Neurosciences, University of California, San Diego, La Jolla, California, San Diego, United States; 4 Genetics and Aging Research Unit, Department of Neurology, Massachusetts General Hospital, Charlestown, Massachusetts, United States; 5 Leonard Davis School of Gerontology, University of Southern California, Los Angeles, California, United States

## Abstract

Air pollution is associated with accelerated cognitive decline and increased risk of dementia in older populations (Cacciottolo et al 2017; Chen et al 2017). Rodent models of air pollution exposure also show Alzheimer-like changes including glial inflammatory responses and increased levels of endogenous amyloid beta (Aβ) peptides (Cacciottolo et al 2017 Levesque et al 2011). We hypothesized that pharmacological inhibition of Aβ production during nPM exposure would attenuate the amyloidogenic processing of APP and glial inflammatory responses. This hypothesis was tested using the γ-secretase modulator (GSM) (BPN-15606) which decreases Aβ42 levels in wild-type rodents (Wagner et al 2017; Kounnas et al. 2010). After nPM exposure of C57BL/6J male mice for 8 weeks, GSM-feeding attenuated pro-amyloidogenic and microglial inflammatory responses. Cerebral cortex levels of Aβ40 and 42 peptides were decreased by 35% and 45% respectively in mice exposed to filtered air and fed with GSM. Hippocampal levels of microglial Iba1 remained at control levels, a decrement of 50% below GSM treated mice. We suggest the Alzheimer candidate drug BPN-15606 has potential benefits to reducing the possible impact of urban air pollution in cognitive aging and Alzheimer risk. The attenuation of pro-amyloidogenic and microglial inflammatory responses is consistent with a role of endogenous Aβ levels in glial inflammatory responses and cognitive impairments from air pollution. Cacciottolo et al 2017, PMID 28140404; Chen H et al. 2017, PMID: 28917207; Levesque et al 2011, PMID 21864400; Wagner et al 2017, PMID 28416568; Kounnas 2010, PMID 20826309 Funding: P01 AG055367, R21AG050201, Cure Alzheimer’s Fund

